# Physiotherapy applied to palliative care patients: a descriptive practice-based study

**DOI:** 10.1186/s12904-023-01188-3

**Published:** 2023-07-20

**Authors:** A Navarro-Meléndez, MJ Gimenez, Y Robledo-Donascimento, A Río-González, A Lendínez-Mesa

**Affiliations:** 1 Rehabilitation area, Hospital Fundación Instituto San José, Avenida de la Hospitalidad s/n, Madrid, 28054 Spain; 2 Department of Health Sciences, “San Juan de Dios” School of Nursing and Physical Therapy, Comillas Pontifical University, Avenida San Juan de Dios, 1, Ciempozuelos, 28350 Spain; 3 Department of Physiotherapy, Faculty of Sport Sciences, Universidad Europea de Madrid, Calle Tajo s/n, Villaviciosa de Odón, 28670 Spain; 4 Hospital Universitario del Henares and Centro de Investigación Fisioterapia y Dolor, Avenida de Marie Curie 0, Coslada, 28822 Spain; 5 Department of Nursing, Neurology Service. Grupo de Investigación en Cuidados (InveCuid), Hospital 12 de Octubre, Avenida de Córdoba s/n, Madrid, 28041 Spain

**Keywords:** MeSH/ DeCS terms: palliative care, Physical therapy, Rehabilitation, Quality of life, Barthel, Functional ambulation categories scale, Palliative Performance Scale, Braden

## Abstract

**Background:**

Over the last few years, the presence of physiotherapists in Palliative Care Units (PCU) has considerably grown based on evidence from studies supporting the use of non-pharmacological measures as part of Palliative Care (PC) treatments. However, more accumulated data are needed to definitively establish its added value. The present study describes the type of patients receiving physiotherapy in a PCU and the benefits obtained in relation to their degree of functional dependence.

**Methods:**

An observational, prospective, descriptive, practice-based study was undertaken involving patients admitted to the PCU of Fundación Instituto San José (Madrid, Spain), who according to the PCU´s clinical practice, met the criteria for physiotherapy intervention. Daily clinical practice was unchanged for study reasons. Participants were assessed prior to initiating and at the end of the physiotherapy program using the following standard scales: the Barthel Index, the Functional Ambulation Categories scale, the Palliative Performance Scale, and the Braden scale. A descriptive analysis was performed and scale scores prior to and after treatment were compared using the Wilcoxon signed-rank test. Significance was set at 0.05.

**Results:**

A total of 63 patients were included (mean age 71.98 ± 12.72; 61.9% males). Fifty-eight patients (92.1%) were oncological patients; of them, 35 (60.3%) had metastases. Prior to treatment, 28 (44.4%) participants had total dependence according to the Barthel index, and 37 (58.7%) were non-functional ambulator according to the FAC scale. At the end of treatment, the number of patients with total dependence decreased to 15 (23.8%) and those non-functional ambulator to 12 (19.0%).

**Conclusions:**

Patients who benefited from physical therapy during their admission to our PCU were predominantly males with oncological processes, mainly lung cancer. PC including physiotherapy improved their functionality, independence and skills for activities of daily living in this sample of PCU patients.

## Background

Rehabilitation and palliative care (PC) are two concepts, which, although they may have been considered disparate due to the patients´ different profiles at which they are aimed, they are both part of the concept universal health coverage of the World Health Organization [1]. From then, advances in research and efforts to improve the quality of care for terminally patients have grown. PC focuses on improving well-being and quality of life in people suffering from a life-threatening illness [2]. Rehabilitation aims to improve the quality of survival through a multimodal person-centred process, and to enhance the functionality of patients, whatever their life expectancy [3]. Physiotherapy, as a discipline that is a vital part of rehabilitation, recovers, maintains or slows down the impact of the disease processes on the patient´s functionality, and is a non-pharmacological therapeutic alternative that complements medical treatment at any stage of an illness. That is why both concepts, aimed at increasing the patient´s quality of life, share objectives and complement each other [4].

In patients receiving PC needs, physiotherapy can improve, stabilize or slow down the functional deterioration of the patient resulting from the disease process [5].Control of symptoms and the improvement in the patient’s independence can produce a physical and psychological relief that may improve self-esteem and reduce feelings of frustration and/or neglect [6, 7]. Physiotherapy also has an important role in providing to families or primary caregivers with the necessary tools (i.e., good body mechanics, the use of different equipment, or to assist in transfer) for the optimal management of patients [8]. For this reason, physiotherapy interventions can be provided to the patient throughout the different phases of their disease being, through dynamic assessments that, adapted to the needs that may arise during the disease process in order to maximize efficacy by always applying the best tools.

Over the last few years, several studies have supported the use of non-pharmacological measures as part of PC treatments based on their efficacy [9–13]. Respiratory techniques have been used to reduce dyspnoea [9] or to provide both manual and mechanical tools for the good management of airway secretions [10]. Other studies have evaluated the application of massage [11, 12] on PC patients as a technique to reduce anxiety and pain, thus reducing the number of rescue doses, according to Pedersen [12], directly affecting and improving well-being in patients receiving PC needs [14]. Programs of therapeutic exercise for patients with advanced cancer have also demonstrated their effectiveness mainly in improving fatigue [9], mood, pain, independence and in reducing workload of caregivers and family members [13], thus optimizing the quality of life for all [13, 14].

In recent years, the inclusion of physiotherapy interventions among other treatments applied in the PC unit (PCU) has considerably grown. A study exploring its application, such as of the one by Orts Candela [15] found that only 1.96% of patients admitted to a PCU received physiotherapy, with all being oncological patients. Thanks to clinical experience and literary evidence about the application of physiotherapy in palliative patients, now also including non-oncological profiles (e.g. neuromuscular diseases) [16], in our centre, up to 15% of total admissions to the PCU receive physiotherapy assessment and interventions. This growth could be interpreted as a consequence of considering physiotherapy as a key element in PC teams.

However, more evidence on the benefits of physiotherapy in this field is still needed to generalise physiotherapy in the multidisciplinary approach to the patient with PC needs. In particular, in order to plan allocations, it is important to provide objective data from studies describing the benefits of physiotherapy in the PC setting. The main objective of this study was to investigate the typology of patients who received physiotherapy in a PCU and the benefits obtained with the palliative care including physiotherapy in relation to their degree of functional dependence.

## Methods

### Study design

An observational, prospective, descriptive, practice-based study was carried out involving patients admitted to the PCU of the Rehabilitation Department of Fundación Instituto San José (FISJ), Madrid, Spain during the period from January to December 2020. The PCU of FISJ is a 30-bed centre where inpatient PC is provided by a multidisciplinary team including 2 physicians specialized in palliative care and 4 nurses; it also has the support of 1 physical and rehabilitation medicine physician, 6 nursing assistants, 1 psychologist, 1 social worker, 1 chaplain and 2 physiotherapists. Patients, referred from other Community of Madrid´s hospitals, were assessed on arrival by a multidisciplinary team to establish the initial pharmacological treatment to control pain and other possible symptoms/signs such as anorexia, constipation, vomiting or dyspnoea. The physical medicine and rehabilitation physician decided, after assessment, to refer patients to the physiotherapy service if the patient met the criteria for physiotherapy. These criteria were clinical stability of the patients (i.e., control of symptoms), and had some aspects of their condition that were considered as potentially reversible, and excluding patients in the stages of pre-mortem phase. Throughout their stay in the PCU, the patients continued to receive pharmacological treatment to maintain symptomatic stability. For the development of this study, the standard practice established since the creation of the Physiotherapy Service in the PCU was followed, without modifications in the criteria for the addition of physiotherapy to the pharmacological PC. The identification of patients who could benefit from physiotherapy or the techniques to be used were, as usual, individually analysed taking into account their characteristics and needs of every single patient. Physiotherapy treatment was carried out in the physiotherapy room of the PCU. The physiotherapy techniques applied to the patients were therapeutic exercises, passive mobilisations, relaxation techniques, respiratory physiotherapy techniques and analgesic therapies. Each day, the physiotherapist individually adapted the type of techniques used to each patient’s situations, without a predefined treatment protocol. Five one-hour sessions per week were assigned to each patient.

This study was carried out in accordance with the principles of the Declaration of Helsinki and the protocol was approved by the Ethics Committee of the Hospital Clínico San Carlos in Madrid on February 20, 2020 with code 20/045-E Thesis. Informed consent was obtained in writing from patients or relatives prior to initiating physiotherapeutic procedures.

### Sampling

Data from all patients referred to physiotherapy by the physical and rehabilitation medicine physician of the FISJ, who were aged between 16 and 99 years old, clinically stable, with sufficient trunk control to remain seated for at least one hour (duration of the physiotherapy session) and who had expressed their willingness to receive physiotherapy treatment were included in the study. Patients were treated during their stay in the PCU and the reasons for the cessation of physiotherapy treatment (e.g., hospital discharge, clinical worsening or death) were recorded.

### Data collection

Demographic data (age, gender), main diagnosis, presence of metastasis in cancer patients, comorbidities or habits (e.g., diabetes, hypertension, smoking or alcoholism), signs and symptoms, physiotherapy techniques applied, duration of treatment periods, and reason for cessation of physiotherapy treatment were recorded for all patients included.

Patients were assessed daily by medical doctors and physiotherapists. The following standardised scales, which are those routinely used in the PCU, were measured once per week to objectively assess the evolution of patients: the Barthel Index [17], the Functional Ambulation Categories (FAC) scale [18], the Palliative Performance Scale (PPS) [19] and the Braden scale [20].

The Barthel Index measures degree of functional dependence in activities of daily living such as feeding, toileting, sphincter control and walking (ranging from 0 to 100; 0 the worst) [17]. The FAC scale measures degree of functional walking (ranging from 0 to 5; 5 the best) [18]. The PPS measures functional performance and estimate survival of patients with terminal illnesses (ranging from 0 to 100; 100 the best) [19]. The Braden scale predicts and calculates the risk of pressure ulcers according to the state of the skin, functionality, and nutritional status of the patient (ranging from 6 to 23; 23 excellent) [20, 21].

For the present study, measured data were collected at 2 time points: prior to initiating and at the end of the physiotherapy program.

The statistical analysis of data was performed using the IBM SPSS statistics 25 program. Overall and by outcome category (death, clinical worsening, hospital discharge) descriptive analysis was performed for all variables using absolute and relative frequencies for qualitative variables and mean and standard deviation or median and interquartile range for quantitative variables. Comparisons between scale scores prior to and after treatment were performed using the Wilcoxon signed-rank test for non-normal distribution of continuous variables or ordinal variables.

A significance level of 0.05 was maintained for the entire analysis.

## Results

A total of 63 patients were included during the study period. Table 1 shows the demographic characteristics and diagnoses of the study patients. Of the 63 patients included, 58 (92.1%) were subjects diagnosed with an oncologic process; of them, 35 (60.3%) had metastases (Table 1).

**Table 1:** Total and by outcome category Descriptive and Demographic data of patients included in the study

	Total (n = 63)	Death (n = 22)	Clinical worsening (n = 23)	Hospital discharge (n = 18)
**Gender**				
Male	39 (61.9)	16 (72.7)	12 (52.2)	11 (61.1)
Female	24 (38.1)	6 (27.3)	11 (47.8)	7 (38.9)
**Age** (mean ± SD)	71.98 ± 12.72	68.18 ± 10.87	74.39 ± 12.71	73.56 ± 14.36
30–39	1 (1.6)	1 (4.5)	0 (0.0)	0 (0.0)
40–49	2 (3.2)	1 (4.5)	0 (0.0)	1 (5.6)
50–59	8 (12.7)	1 (4.5)	4 (17.4)	3 (16.7)
60–69	10 (15.9)	7 (31.8)	2 (8.7)	1 (5.6)
70–79	21 (33.3)	9 (40.9)	7 (30.4)	5 (27.8)
80–89	16 (25.4)	3 (13.6)	8 (34.8)	5 (27.8)
90–99	5 (7.9)	0 (0.0)	2 (8.7)	3 (16.7)
**Main diagnostic**				
Lung cancer	18 (28.6)	8 (36.4)	6 (26.1)	4 (22.2)
Colon and rectal cancer	6 (9.5)	1 (4.5)	2 (8.7)	3 (16.7)
Endometrial cancer	4 (6.3)	0 (0.0)	3 (13.0)	1 (5.6)
Liver cancer	3 (4.8)	0 (0.0)	2 (8.7)	1 (5.6)
Melanoma	3 (4.8)	1 (4.5)	1 (4.3)	1 (5.6)
Pancreatic cancer	3 (4.8)	2 (9.1)	0 (0.0)	1 (5.6)
Hodgkin´s lymphoma	2 (3.2)	1 (4.5)	1 (4.3)	0 (0.0)
Prostate cancer	2 (3.2)	0 (0.0)	1 (4.3)	1 (5.6)
Leukemia	1 (1.6)	0 (0.0)	0 (0.0)	1 (5.6)
Other Cancers	16 (25.4)	5 (22.7)	7 (30.4)	2 (11.1)
COPD	3 (4.8)	2 (9.1)	0 (0.0)	1 (5.6)
Neuromuscular diseases	1 (1.6)	0 (0.0)	0 (0.0)	1 (5.6)
Parkinson’s disease	1 (1.6)	0 (0.0)	0 (0.0)	1 (5.6)
**Metastasis**				
Liver	12 (19.0)	3 (13.6)	6 (26.1)	3 (16.7)
Bones	11 (17.5)	6 (27.3)	2 (8.7)	3 (16.7)
Pulmonary	10 (15.9)	3 (13.6)	5 (21.7)	2 (11.1)
Brain	9 (14.3)	5 (22.7)	1 (4.3)	3 (16.7)
Ganglionic	6 (9.5)	3 (13.6)	2 (8.7)	1 (5.6)
Other	5 (7.9)	3 (13.6)	2 (8.7)	0 (0.0)
No metastasis	28 (44.4)	7 (31.8)	12 (52.2)	9 (50.0)
**Comorbidities and habits**				
Hypertension	29 (46.0)	4 (18.2)	16 (69.6)	9 (50)
Diabetes	25 (39.7)	7 (31.8)	11 (47.8)	7 (38.9)
Dyslipidemia	12 (19.0)	1 (4.5)	6 (26.1)	5 (27.8)
Smoking	5 (7.9)	2 (9.1)	1 (4.3)	2 (11.1)
Obesity	3 (4.8)	0 (0.0)	1 (4.3)	2 (11.1)
Alcoholism	0 (0.0)	0 (0.0)	0 (0.0)	0 (0.0)
Other	17 (27.0)	5 (22.7)	9 (39.1)	3 (16.7)
No previous history	16 (25.4)	8 (36.4)	3 (13.0)	5 (27.8)

Data are shown as n (%) except where indicated.

All 63 participants had muscle weakness, accompanied by dyspnoea in 12 (19.0%) participants, fatigue in 1 (1.6%), and secretions in 7 (11.1%); a total of 15 (23.8%) participants had severe pain and 2 (3.2%) had suffered a bone fracture. All study participants received therapeutic exercise, 10 (15.9%) received passive mobilisation, 7 (11.1%) analgesic therapy with electrostimulation (TENS), 7 (11.1%) respiratory physiotherapy and 3 (4.8%) relaxation techniques. Forty (63.5%) participants received therapeutic exercise exclusively, 6 (9.5%) received a combination of therapeutic exercise and respiratory physiotherapy, 6 (9.5%) were treated with therapeutic exercise and analgesic techniques, 6 (9.5%) received passive mobilisation and therapeutic exercise, 2 (3.2%) were treated with passive mobilisation, relaxation techniques and therapeutic exercise, 1 (1.6%) received relaxation techniques and therapeutic exercise, 1 (1.6%) received respiratory physiotherapy, passive mobilisation and therapeutic exercise and 1 (1.6%) received passive mobilisation, analgesic techniques, relaxation techniques and therapeutic exercise.

Participants remained in the PCU for a total (median, RIQ) of 27 (17–42) days, and received a median (RIQ) of 19.0 (13.5–30.0) sessions of physiotherapy. Reasons for cessation of treatment were death in 22 (34.9%) participants, clinical worsening in 23 (36.5%) participants and hospital discharge due to clinical stabilisation in 18 (28.6%) participants.

Table 2 shows the degree of functional dependence in activities of daily living assessed using the Barthel index prior to initiating physiotherapy and at the end of physiotherapy treatment. The median (RIQ) Barthel scale score at the start of physiotherapy was 25.0 (10.0–45.0) corresponding to severe dependency, while at the end of physiotherapy, patients scored 35.0 (20.0–60.0) (p < 0.001) also corresponding to severe dependency (≥ 40 means partially dependent). Of the 63 participants, 28 (44.4%) patients were totally dependent prior to treatment, while at the end of treatment this decreased to 15 (23.8%) (p < 0.001). This reduction of dependency was evidenced in all outcome categories.

**Table 2:** Total and by outcome category Degree of dependency assessed using the Barthel index

	Total (n = 63)		Death (n = 22)		Clinical worsening (n = 23)		Hospital discharge (n = 18)	
	PRE-PT	POST-PT	PRE-PT	POST-PT	PRE-PT	POST-PT	PRE-PT	POST-PT
**Total**	28 (44.4)	15 (23.8)	9 (40.9)	6 (27.3)	10 (43.5)	7 (30.4)	9 (50.0)	2 (11.1)
**Serious**	14 (22.2)	16 (25.4)	4 (18.2)	5 (22.7)	7 (30.4)	7 (30.4)	3 (16.7)	4 (22.2)
**Moderate**	13 (20.6)	15 (23.8)	5 (22.7)	7 (31.8)	5 (21.7)	6 (26.1)	3 (16.7)	2 (11.1)
**Mild**	7 (11.1)	16 (25.4)	3 (13.6)	3 (13.6)	1 (4.3)	3 (13.0)	3 (16.7)	10 (55.6)
**Independent**	1 (1.6)	1 (1.6)	1 (4.5)	1 (4.5)	0 (0.0)	0 (0.0)	0 (0.0)	0 (0.0)

PRE-PT: Prior to physiotherapy; POST-PT: End of physiotherapy. Data are shown as n (%).

Table 3 shows the degree of functional walking using the FAC scale. Prior to initiate physiotherapy treatment, 37 out of the 63 (58.7%) participants had a score of 0 on the scale (Non-functional ambulatory), a figure that decreased to 12 (19%) at the end of treatment (p < 0.001). An improvement in the functional capacity of participants was observed regardless the outcome category.

**Table 3:** Total and by outcome category Degree of functionality of the gait assessed using the FAC scale

Category	Total (n = 63)		Death (n = 22)		Clinical worsening (n = 23)		Hospital discharge (n = 18)	
	PRE-PT	POST-PT	PRE-PT	POST-PT	PRE-PT	POST-PT	PRE-PT	POST-PT
**0**	37 (58.7)	12 (19)	11 (50.0)	5 (22.7)	17 (73.9)	5 (21.7)	9 (50.0)	2 (11.1)
**1**	19 (30.2)	25 (39.7)	6 (27.3)	11 (50.0)	6 (26.1)	12 (52.2)	7 (38.9)	2 (11.1)
**2**	4 (6.3)	4 (6.3)	4 (18.2)	0 (0.0)	0 (0.0)	2 (8.7)	0 (0.0)	2 (11.1)
**3**	0 (0.0)	10 (15.9)	0 (0.0)	2 (9.1)	0 (0.0)	4 (17.4)	0 (0.0)	4 (22.2)
**4**	3 (4.8)	10 (15.9)	1 (4.5)	4 (18.2)	0 (0.0)	0 (0.0)	2 (11.1)	6 (33.3)
**5**	0 (0.0)	2 (3.2)	0 (0.0)	0 (0.0)	0 (0.0)	0 (0.0)	0 (0.0)	2 (11.1)

PRE-PT: Prior to physiotherapy; POST-PT: End of physiotherapy. Data are shown as n (%). Score: 0 (Non-functional ambulator); 1 (Ambulator, dependent on physical assistance – level I); 2 (Ambulator, dependent on physical assistance – level II); 3 (Ambulator, dependent on supervision); 4 (Ambulator, independent level surface only) and 5 ( Ambulator, independent)

The mean (± SD) pressure ulcer risk score before the start of physiotherapy was 15.73 ± 3.07, indicating a low risk. At the end of treatment, the mean score was 16.52 ± 3.60, maintaining a low risk of pressure ulcer occurrence (p = 0.036). The risk of pressure ulcer development (Braden Scale) in the 63 participants before the start of physiotherapy was 41 (65.1%) participants at low risk, 20 (31.7%) participants at moderate risk and 2 (3.2%) participants at high risk. At the end of treatment, 43 (68.3%) participants were at low risk, 17 (27%) participants at medium risk and 3 (4.8%) participants at high risk (p = 0.835).

Regarding the palliative functionality grade (Table 4), before the physiotherapy intervention, 16 (25.5%) participants had an initial palliative functionality grade score < 40 (i.e., mainly in bed and completely dependent), 34 (53.9%) participants had a score of 50 (i.e., mainly seated and dependant) and 13 (20.6%) participants scored > 60 (i.e., reduced to full ambulation, not requiring assistance or only occasionally). After completion of treatment, 17 (27.0%) participants scored < 40, 21 (33.3%) participants scored 50 and 25 (39.7%) participants scored > 60, (p = 0.016).

**Table 4:** Total and by outcome category Degree of palliative functionality using the PPS scale

	Total (n = 63)		Death (n = 22)		Clinical worsening (n = 23)		Hospital discharge (n = 18)	
%	**PRE-PT**	**POST-PT**	**PRE-PT**	**POST-PT**	**PRE-PT**	**POST-PT**	**PRE-PT**	**POST-PT**
**0**	0 (0.0)	0 (0.0)	0 (0.0)	0 (0.0)	0 (0.0)	0 (0.0)	0 (0.0)	0 (0.0)
**10**	0 (0.0)	3 (4.8)	0 (0.0)	2 (9.1)	0 (0.0)	1 (4.3)	0 (0.0)	0 (0.0)
**20**	2 (3.2)	1 (1.6)	1 (4.5)	1 (4.5)	0 (0.0)	0 (0.0)	1 (5.6)	0 (0.0)
**30**	3 (4.8)	1 (1.6)	1 (4.5)	0 (0.0)	1 (4.3)	1 (4.3)	1 (5.6)	0 (0.0)
**40**	4 (6.3)	12 (19.0)	2 (9.1)	5 (22.7)	2 (8.7)	7 (30.4)	0 (0.0)	0 (0.0)
**50**	43 (68.3)	21 (33.3)	14 (63.6)	10 (45.5)	17 (73.9)	9 (39.1)	12 (66.7)	2 (11.1)
**60**	9 (14.3)	17 (27.0)	3 (13.6)	1 (4.5)	3 (13.0)	5 (21.7)	3 (16.7)	11 (61.1)
**70**	2 (3.2)	3 (4.8)	1 (4.5)	1 (4.5)	0 (0.0)	0 (0.0)	1 (5.6)	2 (11.1)
**80**	0 (0.0)	4 (6.3)	0 (0.0)	2 (9.1)	0 (0.0)	0 (0.0)	0 (0.0)	2 (11.1)
**90**	0 (0.0)	1 (1.6)	0 (0.0)	0 (0.0)	0 (0.0)	0 (0.0)	0 (0.0)	1 (5.6)
**100**	0 (0.0)	0 (0.0)	0 (0.0)	0 (0.0)	0 (0.0)	0 (0.0)	0 (0.0)	0 (0.0)

PRE-PT: Prior to physiotherapy; POST-PT: End of physiotherapy. Data are shown as n (%). The percentage of 0% means that the patient is deceased and the score of 100% means that the patient is fully ambulatory and healthy.

## Discussion

This study was carried out to analyse the profile of patients under PC who received physiotherapy intervention during their stay in our PCU: their characteristics, the intervention techniques used, and the effect of these interventions observed. The data showed that, in our centre, patients who received physiotherapy treatment were mainly males diagnosed with oncological processes. Overall, an improvement in the patient’s situation (i.e., dependency, and functionality) was seen regardless of the reason for physiotherapy treatment being ceased (i.e., hospital discharge, clinical worsening or death).

Lung cancer was the most frequent cancer, a fact which is in accordance with worldwide epidemiological data, since despite having been surpassed in number of cases by breast cancer, the most common cancer nowadays [22], lung cancer continues to be responsible for the greatest number of deaths [22]. Slightly more than half of the subjects presented metastases, typical of cancer processes in very advanced stages. Likewise, the most frequent comorbidities among our patients, hypertension and diabetes, are also among the most prevalent in the general population in our country (42.6% and 13.8% respectively) [23]. Based on this, it could be hypothesised that study participants were representative of the population in Spain receiving PC.

The analysis of the degree of dependency measured using the Barthel index showed a statistically significant decrease in the percentage of totally dependent patients (from 44.4 to 23.8%) and an increase in patients with mild dependency (from 11.1 to 25.4%), suggesting a benefit from PC including physiotherapy. These results are in line with other studies confirming that rehabilitation/physiotherapy and physical exercise intervention programmes improve the degree of dependency [24, 25]. This improvement was also reflected in gait functionality, as almost 60% of the patients were unable to walk or needed great help to walk prior to commencement of the intervention, whereas the number of people unable to walk dropped to 19% at the conclusion of the intervention. This improvement was observed in all outcome categories, but especially in those who were finally discharged from the PCU and returned home. According to these data, the functional decline experienced by this type of patients due to prolonged hospitalization (i.e., primarily functional impairment, loss of muscle strength, pain, fatigue and oedema [26]), the main reason for referral to a specialized PCU, can be partially reversed by adequate care. This confirms previous reports showing that referral of patients to specialised PCUs that include physiotherapy interventions can considerably improve the patient’s survival rates, functionality, independence and skills for activities of daily living [27, 28]. Therefore, a progressively applied and individualized therapeutic exercise programme, together with other techniques such as massage, compressive bandages or analgesic electrotherapy [29], seems able to improve the patient’s conditions even in the PC setting. This improvement in the functionality of the palliative patient implies not only individual benefits for the patient but also financial savings for healthcare institutions [28]. However, despite these benefits and patient demands [30], maintaining physical and functional fitness is often not a priority in PCUs [31]. This is because the traditional PC concept is more focused on satisfying the patient’s emotional needs (assuring the patient’s wishes for a good death) over functionality, which is not recognised as essential, even though it is one of the main wishes for a high number of patients.

Although to a lesser extent, the risk of pressure ulcers (assessed by the Braden scale) also improved in our patients. This minor improvement could be related to a published meta-analysis which showed that the Braden scale has moderate predictive validity and low predictive specificity for pressure ulcers in long-term care residents [21].In patients with advanced diseases, it is considered that the information collected through the PPS scale could replace the use of the Braden scale [32]. In our study, patients with palliative functional status (assessed through the PPS scale) also showed a positive evolution: at baseline almost 70% of patients required “mainly in bed”, decreasing to 19% at the end of the physiotherapy treatment, with an improvement in the groups “mainly sit/lie” or “reduced ambulation”.

The limitations of our study are mainly based on ethical reasons that limit our choice of study designs, since observational cohort studies or experimental studies (including non-treated control groups with physiotherapy) could definitively assess the added value of physiotherapy. As in any study based on clinical practice, there was no homogeneity in the profiles of the patients included in the study since all patients that, both the PC specialists and the rehabilitation team considered that could benefit from physiotherapy treatment, were included after signature of the informed consent. This lack of selection of participants implied that subjects differed both in age and in main diagnosis or stage of disease. The techniques used for treatment or the duration of the sessions were also heterogeneous since they were adapted according to the individual needs of the patients. Similarly, the number of sessions and duration of physiotherapy were also not pre-established but always tailored to the daily patient’s situation, as occurs in the routine practice in the PCU. This study was limited to the assessment of functionality and daily life activities, important objectives of physical therapy, and did not evaluate the evolution of other symptoms as dyspnoea or pain mainly controlled by pharmacological treatment. Another limitation was the emergency situation created by the pandemic SARS-CoV-2 in March 2020 which altered the functioning of our unit, as the admission of patients to the rehabilitation service was suspended. The facilities were used for other purposes and healthcare professionals were reorganised to meet other care needs. This hiatus in the operation of the unit reduced the number of subjects for the study. However, the strength of our study was the evaluation of PC including physiotherapy practice in a real-life setting of a PCU, taking into account the individualised needs of patients and the functioning of the unit. Furthermore, standardised scales were used to objectively assess results, as in previous publications in the rehabilitation field. This reduced the risk derived from the non-blinded assessment of participants. In this sense, to our knowledge no previous studies have showed the benefit of combining PC with physiotherapy in palliative patients using the PPS scale as in the present study, and the existing literature is scattered with respect to the profiles of patients who benefit from non-pharmacological therapies in PCU [11]. The efficacy of physiotherapy in patients with lung cancer, the most diagnostic group in our study, through therapeutic exercise, rehabilitation and early PC has been reported, with significant improvements in functional and physical capacities, muscle strength and quality of life, as well as in dyspnoea in countries such as Spain, Germany and Belgium [33–35]. The present study adds to the literature a considerable number of patients evaluated during a long time of period, and analysing data using objective measures.

## Conclusion

The present study showed that, in our centre, patients who benefited from the provision of physiotherapy during their admission to a PCU were predominantly males with oncological processes, mainly lung cancer. PC including physiotherapy improved their functionality, independence and skills for activities of daily living as assessed by the use of standard scales. These results are important to increase awareness of the benefits that physiotherapy may provide to patients affected by terminal illnesses.

## Data Availability

The datasets generated during and/or analysed during the current study are available from the corresponding author on reasonable request.

## List of abbreviations

PC: palliative care
FISJ: Fundación Instituto San José
FAC: Functional ambulation categories
PPS: Palliative Performance Scale
TENS: Transcutaneous electrical nerve stimulation
